# A proof-of-concept experimental-theoretical model to predict pesticide resistance evolution

**DOI:** 10.1038/s41437-025-00781-x

**Published:** 2025-07-23

**Authors:** Luna Qingyang Li, Liisa Parts, Philip Madgwick, Kayla King, Anthony Flemming, Alison Woollard

**Affiliations:** 1 Department of Biochemistry, University of Oxford, Oxford, UK; 2 Syngenta, Jealott’s Hill International Research Centre, Bracknell, UK; 3 Department of Zoology, University of British Columbia, Vancouver, Canada; 4 Department of Microbiology & Immunology, University of British Columbia, Vancouver, Canada; 5 Department of Biology, University of Oxford, Oxford, UK

## Abstract

Insecticide resistance poses a major challenge to sustainable agriculture, yet studying its evolution in laboratory settings is notoriously difficult due to challenges related to maintaining large populations of pest species. While theoretical models offer valuable predictions, an experimental system for validating insecticide resistance management strategies remains lacking. Here, we explore *C. elegans* as a model organism for studying insecticide resistance evolution. We developed an in silico population genetics model and tested its predictive power in laboratory experiments, comparing the computational predictions to experimental resistance selection dynamics. Two compounds with distinct modes of action were tested to assess the generalizability of this system across different resistance mechanisms. Our results showed that in silico predictions generally resembled multigenerational in vivo resistance selection outcomes, demonstrating the feasibility of integrating in vivo and in silico modelling approaches in resistance research. By bridging the gap between theoretical and empirical research, this framework paves the way for addressing a wide range of open questions in resistance management, permitting the development of better informed and more effective resistance management strategies for the agricultural industry.

## Introduction

Managing crop losses due to arthropod pests is one of the chief challenges facing the global agricultural industry (Gould et al. 2018; Mateos Fernandez et al. 2022). Since the invention of synthetic chemical insecticides in the 1940s (Casida and Durkin 2017), their ease of production and field application have led to widespread use (Siddiqui et al. 2022). Although pesticides are an effective solution when initially deployed, arthropods, like human pathogens, are highly adaptable in the face of chemical selection. To date, over 15,000 cases of arthropod pesticide resistance have been reported in over 600 species (Arthropod Pesticide Resistance Database, www.pesticideresistance.org/). Better understanding of pest control and resistance management is therefore of vital importance for ensuring global food security.

Resistance evolution in the field consists of two phases: the emergence phase and the selection phase (Hobbelen et al. 2014; Madgwick et al. 2024). Existing work in the area concentrates on resistance phenotypes in the selection phase where resistance has already spread in a population. A broad literature exists to illustrate the genetic and transcriptomic underpinnings of field-observed pesticide resistance (Georghiou 1972; Catania et al. 2004; Watson et al. 2010; Troczka et al. 2012; Sammons and Gaines 2014; Bass et al. 2015; Lucas et al. 2015; Omrane et al. 2015; Gaines et al. 2020; Zhu et al. 2021; Siddiqui et al. 2022; Yin et al. 2023). Theoretical modelling work since the 1960s has led to recommendations of various resistance management regimes such as the use of mixtures and rotations (Kimura 1962; Comins 1977; Curtis 1985; Mani 1985; Comins 1986; May 1986; Roush 1989; Tabashnik 1989; REX-Consortium 2013; Levick et al. 2017; Madgwick and Kanitz 2022a, 2022b, 2024). However, empirical evidence supporting theoretical insights has lagged behind. Recently, experimental evolution studies have been conducted to evaluate resistance management strategies in certain laboratory populations typically for fungicide and herbicide resistance (McKenzie and Batterham 1998; Prabhaker et al. 1998; Neve and Powles 2005; Parker et al. 2006; Lagator et al. 2013; Jacomb et al. 2016; Ballu et al. 2021; Zoh et al. 2021; Sadia et al. 2024). Given that pesticide resistance frequency will continue to rise, now more than ever before there is a need to take an interdisciplinary approach to enable not just the description but also accurate prediction of resistance evolution under different management approaches.

The prediction of pesticide resistance evolution will depend on the type of organism involved. Fungi, weeds, and insects exhibit fundamentally distinct patterns of resistance evolution upon encountering pesticidal or herbicidal chemicals. According to a comprehensive survey conducted by Hawkins et al. (Hawkins et al. 2019), fungicide resistance is commonly found as de novo targetsite mutations, herbicide resistance tends to rely on standing variation in compound metabolism, whereas insecticide resistance evolution displays characteristics of both of these mechanisms. For fungicide and herbicide resistance, model organisms have been developed in which large laboratory populations can be maintained and resistance evolution can be observed within a relatively short time frame (Roux et al. 2005; Lagator et al. 2013; Ballu et al. 2021). This is not the case for insecticide resistance. Pest insect species are not naturally well-suited to laboratory evolution experiments: it is difficult to maintain sufficiently large populations which are minimally affected by genetic drift in the laboratory, and generation time is usually long, preventing rapid iteration. Existing work on experimentally evolving insect populations of *Anopheles gambiae* (Zoh et al. 2021; Sadia et al. 2024), *Myzus persicae* (Parker et al. 2006), and *Tribolium castaneum* (Jacomb et al. 2016) involved population sizes in the low tens to the low hundreds in a single replicate line. Therefore, while working in pests offers a realistic experimental system from a pharmacological perspective, these systems lack efficacy in evolutionary experiments because they cannot be cultured at sufficient scale, both in terms of number of individuals and number of generations.

To address this, we considered potential model or surrogate systems for studying resistance evolution. One possible model is *Drosophila melanogaster. Drosophila* species have been successfully used in mechanistic studies of pesticide resistance (Catania et al. 2004; Watson et al. 2010; Le Goff and Hilliou 2017; Seong et al. 2019), and *Drosophila* was used as a model organism for constructing insecticide resistance-reversing gene drives (Kaduskar et al. 2022). However, it remains the case that generation time is in the order of weeks (Ashburner 1978; Ashburner et al. 2005), and typical population sizes are in the range of hundreds. We concluded that this system would sacrifice pharmacological applicability for insufficient gains in scalability.

In search of another suitable model organism for the study of insecticide resistance evolution, we turned to the nematode genus *Caenorhabditis* and the model species *C. elegans*. This species is small (1 mm long), has a short 3–4 day lifecycle (Muschiol et al. 2009), and can be readily cultured in the laboratory at larger scales than insects – tens of thousands of animals can be maintained with ease. Furthermore, there are features of the *C. elegans* system and resources of the associated research field that are useful in pesticide resistance studies. A large strain collection exists and tens of thousands of distinct phenotypes have annotated genetic basis, both of which provide invaluable resource for understanding both the mechanism and the evolutionary dynamics of resistance. *C. elegans* is highly amenable to genetic manipulation (Dickinson and Goldstein 2016; Kim and Colaiacovo 2019), and there are well established protocols to quantify life-history traits and fitness (Braendle and Paaby 2024), facilitating strain fitness characterisation. Importantly, discrete, non-overlapping generations (~2 per week) can be created using the population bleaching technique (Porta-de-la-Riva et al. 2012). Each passage involves dissolving egg-bearing adults in bleach leaving only the embryos intact as these are protected by chitin (Johnston and Dennis 2012). This is convenient for cross-comparison with in silico modelling outcomes, as population genetics models – a widely used theoretical framework for simulating evolutionary dynamics (Levick et al. 2017; Madgwick and Kanitz 2022a) – typically operates on the assumption of discrete generations. If required, ancestral strains can be cryopreserved to provide direct comparison between pre- and post-evolved populations.

We concluded that *C. elegans* has attractive features as an experimental tool for studying the evolution of resistance. While evolution is biologically universal, *C. elegans* is not an insect and a possible concern is that it would not adequately reflect relevant pest pharmacology to yield informative insights into pesticide resistance evolution. However, *C. elegans* has a successful track record in the study of pesticide pharmacology which greatly mitigates this concern. *C. elegans* have been used as a model organism for discovering several insecticide modes-of-action (Sluder et al. 2012; Bian et al. 2018; Dennis et al. 2018; Guest et al. 2020), indicating that there is sufficient homology in its biology to that of field-relevant insects, at least with respect to pesticidal modes of action. Indeed, in one instance an insecticide mechanism of resistance identified in the lab in *C. elegans* was subsequently observed in pest insects in the field (Guest et al. 2020; Lueke et al. 2020). These studies contributed to the current collection of nematode strains carrying stable genetic mutations conferring protection to insecticides and other pesticidal agrochemicals (Dent et al. 2000; Qian et al. 2008; Welz et al. 2011; Ghosh et al. 2012; Sluder et al. 2012; Rufener et al. 2013; Dennis et al. 2018; Hahnel et al. 2018; Guest et al. 2020; Schleker et al. 2022). Moreover, previous studies have illustrated the ability of nematodes in the *Caenorhabditis* genus to rapidly evolve resistance to agrochemicals under laboratory selection (Lopes et al. 2008; Reynolds et al. 2016). Therefore, we considered that there is sufficient evidence to warrant further investigation of the system for resistance evolution research. The combination of scalability with pharmacological relevance, underpinned by the capabilities and resources of a well-developed model organism community facilitate the possibility of *C. elegans* as a promising system for incorporating mechanistic, theoretical, and evolutionary insights to dissect and predict insecticide resistance dynamics.

As a proof-of-concept investigation, we used *C. elegans* to explore how experimental insight can be combined with theoretical modelling results in a resistance evolution context. There are precedents for combining theoretical and experimental data in managing antimicrobial resistance (Pertrungaro 2021). Specifically, we focused on the selection phase where more is currently known using well characterised pesticide resistance-conferring alleles. *C. elegans* was chosen since strains with known resistance-conferring mutations are readily available (Dent et al. 2000; Qian et al. 2008; Welz et al. 2011; Ghosh et al. 2012; Sluder et al. 2012; Rufener et al. 2013; Dennis et al. 2018; Hahnel et al. 2018; Guest et al. 2020; Schleker et al. 2022). Ordinarily resident in decomposing organic matter (Schulenburg and Felix 2017), in its natural state *C. elegans* reproduces through a distinctive androdioecious mating system where hermaphrodites selffertilise to produce offspring, and cross-progeny from male fertilisation is exceedingly rare (Frezal and Felix 2015). However, genetic knockout of a single gene is sufficient to convert *C. elegans* into a dioecious mating animal (Schedl and Kimble 1988; Hu et al. 2019). This means that the model can be used flexibly to mimic the evolutionary dynamics of pests which reproduce through clonal expansion or dioecious mating. In this proof-of-concept work, we chose the former for simplicity, noting differences between selfing and true asexual reproduction in terms of their long-term evolutionary consequences.

First, we show that a panel of geographically diverse wild *C. elegans* isolates display variable natural sensitivities to a range of pesticidal compounds, demonstrating the suitability of using this species as a model for understanding insecticide resistance. Next, strains carrying known genetic mutations conferring pesticide resistance were used to assess the predictability of resistance spread in a population under selection. Baseline dose-survival and fitness data were gathered, and a competitive microevolution assay (the “in vivo model”) was carried out with and without compound selection for a number of discrete generations. A companion population genetics model (the “in silico model”) was developed to predict the dynamics of the in vivo model using only single-generation baseline data. The accuracy of the in silico prediction was then assessed.

The possibility of iterating between in vivo and in silico models in a pesticide resistance evolution context is significant as it opens the door to addressing a wide range of research questions, from understanding fundamental resistance evolution dynamics to optimal pest control and resistance management strategies. *C. elegans* is a convenient model for shedding light on this important topic. We envision that improved practical and theoretical understanding of pesticide resistance evolution will bring benefit across the agricultural sector.

## Material and Methods

### Chemical susceptibility screen

A panel of twenty-four geographically distinct wild *C. elegans* isolates were obtained from the *C. elegans* Genetics Center (CGC, University of Minnesota) (for strain names and geographical origins see S1). Alongside the laboratory-adapted N2 strain, twenty-five strains in total were tested on twenty-eight pesticidal chemicals. A total of 700 strain-compound pairs were tested to give a representative sample across genetic diversity and compound modes-of-action.

Each strain was exposed to each chemical in 24-well plates, with each strain-compound pair assayed three times. In each well, 0.5 ml of nematode growth media (NGM) were added followed by 30 μl of dissolved chemical (in a solvent of 50% isopropanol, 40% water, 10% DMSO) to reach a final concentration of 5 or 50 ppm per ml depending on the compound (for compound concentration in molar form see S2). The wells were then seeded with bacteria, and allowed to dry in the flow hood.

Ten worms at the L1 stage (first larvae stage) were transferred to each well. The plates were then placed in a 20 °C incubator for 4 days. Drug sensitivity in each well was qualitatively characterised on a scale of 1 to 4 according to criteria outlined in Table 1.

The mean of three repeats is displayed. A Kruskal-Wallis test was performed across all strains exposed to the same compound in GraphPad Prism to assess the significance of variation in strain sensitivity.

### Ivermectin dose-survival assay

Ivermectin was first dissolved in isopropanol to a concentration of 1 mg/ml. For the susceptible strain dose-survival assay, this solution was serially diluted 10-fold three times to reach a concentration of 1 μg/ml, then diluted 2-fold once to the final concentration of 500 ng/ml. To produce the ivermectin concentrations used in the assay, the 500 ng/ml stock solution was serially diluted four in one (concentration ×0.8 per dilution) nine times. In a 24-well plate, 9.38 μl of the ivermectin solution was added to 1.5 ml of NGM per well, establishing a gradient of ten ivermectin concentrations ranging from 0.419 ng/ml to 3.125 ng/ml. 9.38 μl of isopropanol solvent was added to the control wells. Four repeats were conducted per ivermectin concentration. After seeding with OP50 bacteria, 100 nematode eggs were pipetted onto each well. The number of mature adult worms in each well was counted after a four-day incubation at 20 °C. Survival in each well was normalised to the mean of the number of mature adult worms in the control condition. The mean survival for each tested concentration was then plotted.

For the resistant strain dose-survival assay, the 1 mg/ml solution was diluted 10-fold once to a concentration of 100 μg/ml. To produce the ivermectin concentrations used in the assay, the 100 μg/ml stock solution was serially diluted 2-fold ten times. In 24-well plates, 7.68 μl of the ivermectin solution was added to 1.5 ml of NGM per well, establishing a gradient of eleven ivermectin concentrations ranging from 0.5 ng/ml to 512 ng/ml. The following protocol is similar to that of the susceptible strain, starting with 100 nematodes eggs in each well, survival was measured as the number of mature adults in each well normalised to the control condition. Mean survival of the four repeats at each tested concentration was then plotted.

An inverse sigmoid curve was fitted to the dose-survival data using R. The *nls* function within the *stats* package was used to find the values of parameters *a* and *b* which minimise the total squared error of the predicted survival (*ŷ*) compared to the experimentally observed survival value (*y*). The inverse sigmoid curve used for predicting survival from compound concentration has the following form:

(1) $$\hat{y}=\frac{1}{1+e^{a(x-b)}}$$

Where *x* represents the base 2 log of the compound concentrations. The parameter *a* dictates the slope of the inverse sigmoid curve and *b* the midpoint.

### Spirotetramat dose-survival assay

Spirotetramat was first dissolved in a solvent made of 50% water and 50% isopropanol to a concentration of 10 mg/ml. Care was taken to ensure that all crystals have dissolved before any serial dilutions were performed. Unlike ivermectin, the same compound concentrations were used in both the susceptible and resistant strain assays. To produce the spirotetramat concentrations used in the assay, the 10 mg/ml stock solution was serially diluted 2-fold ten times. In a 24-well plate, 38.4 μl of the spirotetramat solution was added to 1.5 ml of NGM per well, establishing a gradient of eleven spirotetramat concentrations ranging from 0.25 μg/ml to 256 μg/ml. 38.4 μl of the solvent was added to the control wells. Four repeats were conducted per spirotetramat concentration. After seeding with OP50 bacteria, 100 nematode eggs were pipetted onto each well. The number of mature adult worms in each well was counted after a four-day incubation at 20 °C. Survival in each well was normalised to the mean of the number of mature adult worms in the control condition. The mean survival for each tested concentration was then plotted.

Dose-survival curve fitting was carried out in a similar fashion to the ivermectin dose-survival assays.

### Developmental time assay

Developmental time was measured using egg-to-egg generation time as a proxy. In each well of a seeded 12-well NGM plate, 50 synchronised nematode eggs were added at hour 0. The plates were incubated at 20 °C. Egg-to-egg generation time in each well was measured as the number of hours until the first F1 egg could be observed. 12 repeats were conducted per tested nematode strain.

The Student’s t-test was conducted in R to determine whether developmental time is significantly different between each pair of strains.

### Fecundity assay for competitive microevolution

Single-worm fecundity was measured by recording the total number of eggs laid by a single worm from day 0 to day 4. Any eggs laid after day 4 would not contribute to the genetic makeup of the offspring generation in the competitive microevolution assay, and therefore were not counted in this fecundity assay. On day 0, one or two nematode eggs were pipetted onto each well of a seeded 12-well NGM plate. The plates were incubated at 20 °C or 22.5 °C (a higher temperature was used to compensate for the developmental delay of the ivermectin-resistant JD608 strain, as was the case in the competitive microevolution assay below). The total number of adult worms as well as the total number of eggs laid in each well were counted on day 4, and the number of eggs laid per worm was calculated. 12 repeats were conducted per tested nematode strain.

Student’s t-test was conducted in R to determine whether fecundity per worm is significantly different between each pair of strains.

### Multi-generation competitive microevolution

Multi-generation competitive microevolution were carried out using only homozygote susceptible or homozygote resistant animals. Prior to the first generation, homozygote susceptible and homozygote resistant adult worms were bleached to give synchronised eggs. To begin the first generation, set proportions of susceptible and resistant nematode eggs were mixed and 7500 eggs in the mixture were pipetted onto 9 cm NGM plates. Compound plates were prepared 4 days prior to seeding of worm eggs. Chemicals added in solvent were allowed to dry for 2 days, at which point bacterial food was seeded.

For spirotetramat assays, resistance was initiated at 5% in the population. The susceptible strain used was PD4792 [*myo-2p::GFP* + *pes-10p::GFP* + gut-promoter::*GFP*], acquired from CGC. PD4792 displays a strong pharyngeal GFP signal under fluorescence microscopy. The resistant strain used was SR42 (Guest et al. 2020) which carries an A1559V point mutation in *pod-2*, the target of spirotetramat. Three conditions were tested: no-compound control, 20 μg/ml spirotetramat, and 24 μg/ml spirotetramat. Six replicate lines were established for each condition.

For ivermectin assays, resistance was initiated at 50% in the evolving populations to compensate for the strong fitness defect of the resistant strain under no selection. PD4792 was used as the susceptible strain. JD608, harbouring a triple-deletion genotype in *avr-14, avr-15*, and *glc-1* (Dent et al. 2000), was used as the resistant strain. Since only resistant homozygotes were used throughout the microevolution assay, there was no possibility of the triple-deletion genotype undergoing recombination, hence the genotype was inherited as one unit. Two conditions were tested: no-compound control and 1.4 ng/ml ivermectin. Six replicate lines were established for each condition.

Spirotetramat selection and control were conducted at 20 °C, ivermectin selection and control were conducted at 22.5 °C. All populations were incubated for 4 days. On day four, each plate of worms was thoroughly washed off into a conical tube using M9 solution (Stiernagle 2006). 300 μl of the M9 solution containing worms was plated on an unseeded NGM plate for resistance frequency measurement. Resistance frequencies were recorded under a fluorescence microscope by counting the number of fluorescent (and therefore susceptible) individuals versus the number of non-fluorescent (and therefore resistant) individuals. The remainder of the population in M9 solution underwent population bleaching (Porta-de-la-Riva et al. 2012) to give rise to eggs, of which 7500 were pipetted onto a new NGM plate to begin the next generation of microevolution.

The competitive microevolution assay was carried out for a sufficient number of generations until the measured resistance frequency was very close to or at 100% in the compound-selection conditions.

### Computational modelling

Population genetics models used in pesticide resistance modelling (Levick et al. 2017; Madgwick and Kanitz 2022b) were adapted to accommodate for the experimental setup of the in vivo model. The in silico model relies on Wright-Fisher evolutionary dynamics (Fisher 1930; Wright 1931), and assumes discrete generations as well as a constant population size of 7500. Only homozygote susceptible and homozygote resistant individuals would arise in the in vivo model, hence their aggregate frequencies in the population were tracked by the in silico model. Within each generation, the initial genotype distribution was inherited from the previous generation, with individuals selected to reproduce with probability proportional to their genotype fitness, and a multinomial sampling method was employed to stochastically determine the genetic makeup of the offspring generation (for full model code in R see S3). One hundred stochastic in silico predictions were generated to give a range of predictions for each in vivo model condition tested.

Fitness of the homozygote susceptible (*w_AA_*) and homozygote resistant (*w_aa_*) strains under compound selection were calculated as the product of each strain’s normalised baseline fecundity (*m_AA_, m_aa_*) with its survival under compound selection (*v_AA_, v_aa_*). Utilising data from single-generation fecundity assays, the baseline fecundity of resistant homozygote strains were normalised to the baseline fecundity of the susceptible homozygote strain which was set to 1. For both the susceptible and resistant strains, survival under compound selection was interpolated from the relevant single-generation dose-survival curve data.

(2) $$w_{AA}=m_{AA}\times v_{AA}$$

(3) $$w_{aa}=1\times v_{aa}$$

For a direct visualisation of the accuracy of the in silico model’s prediction, the relative fitness of the resistant homozygote to the susceptible homozygote, $\frac{w_{AA}}{w_{aa}}$ was compared across the in silico and the in vivo models. The relative fitness is related to the selection coefficient, *s*, through the following formula (Haldane 1927):

(4) $$\frac{w_{AA}}{w_{aa}}=1+s$$

The derivation of the relative fitness from the in silico model is straightforward since *w_AA_* and *w_aa_* are explicitly calculated by the in silico model. For the in vivo model, the relative fitness must be derived from the change in resistance frequency across generations. Specifically, the resistance frequency at generation *t, f_t_*, is related to the initial resistance frequency at generation 0 through the relationship:

(5) $$f_{t}=\frac{1}{1+\left[{\left(1+s\right)}^{-t}\times \frac{1-f_{0}}{f_{0}}\right]}$$

Where *f*_0_ represents the resistance frequency at generation 0, prior to selection. The following linear model was fitted between values of *t* and the experimentally observed values of *f_t_*:

(6) $$model\;=Im\left(\log_{10}\left(\frac{f_{t}}{1-f_{t}}\right)\sim t\right)$$

With the fitted gradient, *g*, representing log_10_ (1 + *s*), and the intercept $-\log_{10}\left(\frac{1-f_{0}}{f_{0}}\right)$. Therefore, the selection coefficient, *s*, can be calculated from the gradient of the fitted linear model through:

(7) $$s=10^{g}-1$$

The selection coefficient thus calculated is related to the relative fitness as described in Eq. 4.

## Results

### Natural variation in pesticide resistance of wild *C. elegans* isolates

**Fig. 1:**
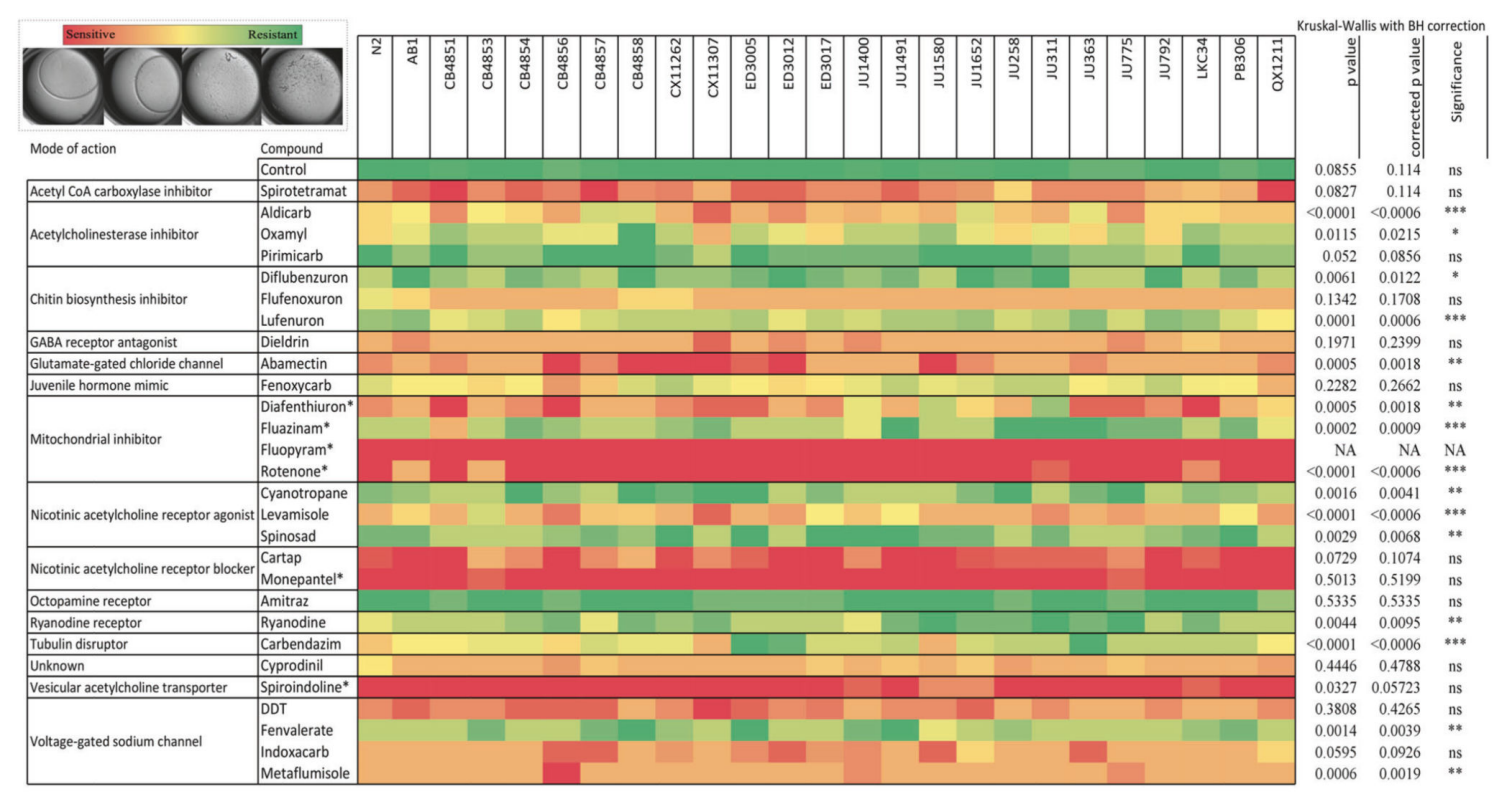
Wild *C. elegans* isolates display natural variation in sensitivity to pesticidal chemicals. A panel of 25 wild *C. elegans* isolates from geographically distant sites were selected. Ten synchronised L1 worms were incubated with 28 different pesticidal compounds at industry-relevant concentrations. On day 4, visual inspection was conducted to determine the sensitivity of the nematode isolate to the chemical compound, with possible values ranging from 1 (lethal) to 4 (unaffected). Compounds annotated with (*) were applied at a concentration of 5 parts per million, with the remaining compounds applied at 50 parts per million. A Kruskal-Wallis test followed by Benjamini-Hochberg correction was conducted in GraphPad Prism to determine if there was a statistically significant difference between mean isolate sensitivities to each compound (**** = *p* < 0.0001, *** = *p* < 0.001, ** = *p* < 0.01, * = *p* < 0.05, ns = *p* > 0.05). The heatmap displays the mean of 3 biological replicates.

Natural variation in resistance phenotype is a prerequisite for developing high-level genotypic resistance. For *C. elegans* to be a suitable model for understanding pesticide resistance evolution, we sought to demonstrate widespread variation in pesticide sensitivity in geographically-distinct wild isolates. Twenty-four well-established isolates, as well the laboratory-adapted N2 strain, were exposed to 28 commercial pesticidal compounds, and susceptibility was rated qualitatively on a scale of 1 (lethal) to 4 (unaffected). Of the 28 compounds tested, 16 compounds displayed a significant difference in strain susceptibility (Fig. 1). Thus, variation in sensitivity to pesticides is widespread among geographically-distinct wild *C. elegans* isolates, suggesting that *C. elegans* has the potential to evolve resistance to a diverse range of pesticidal compounds.

### Pesticide dose-response profiles of susceptible and resistant *C. elegans* strains

Next, we selected two pesticide resistant *C. elegans* strains reported in the literature to investigate in detail: a spirotetramat-resistant strain SR42 which contains a A1559V point mutation in the spirotetramat target gene *pod-2*, encoding the acetyl-CoA carboxylase enzyme, discovered through forward genetics (Guest et al. 2020), and an ivermectin-resistant strain JD608 which contains deletion mutations in the genes encoding the ivermectin-sensitive glutamate-gated ion channel subunits *avr-14, avr-15*, and *glc-1* (Dent et al. 2000). Importantly, resistance to spirotetramat and ivermectin are known to be field-relevant: mutations in the *pod-2* homologue have been observed in wild populations of whiteflies (*Bemisia tabaci*) (Lueke et al. 2020), and ivermectin resistance have been detected in a wide range of pest species ranging from ticks to ectoparasites to parasitic nematodes (Martin et al. 2021; Shyma et al. 2021; Furnival-Adams et al. 2024). Spirotetramat and ivermectin exhibit distinct modes-of-action and are both widely applied pesticidal agents respectively against insects and helminths respectively (Laing et al. 2017; Gutbrod et al. 2020).

**Fig. 2:**
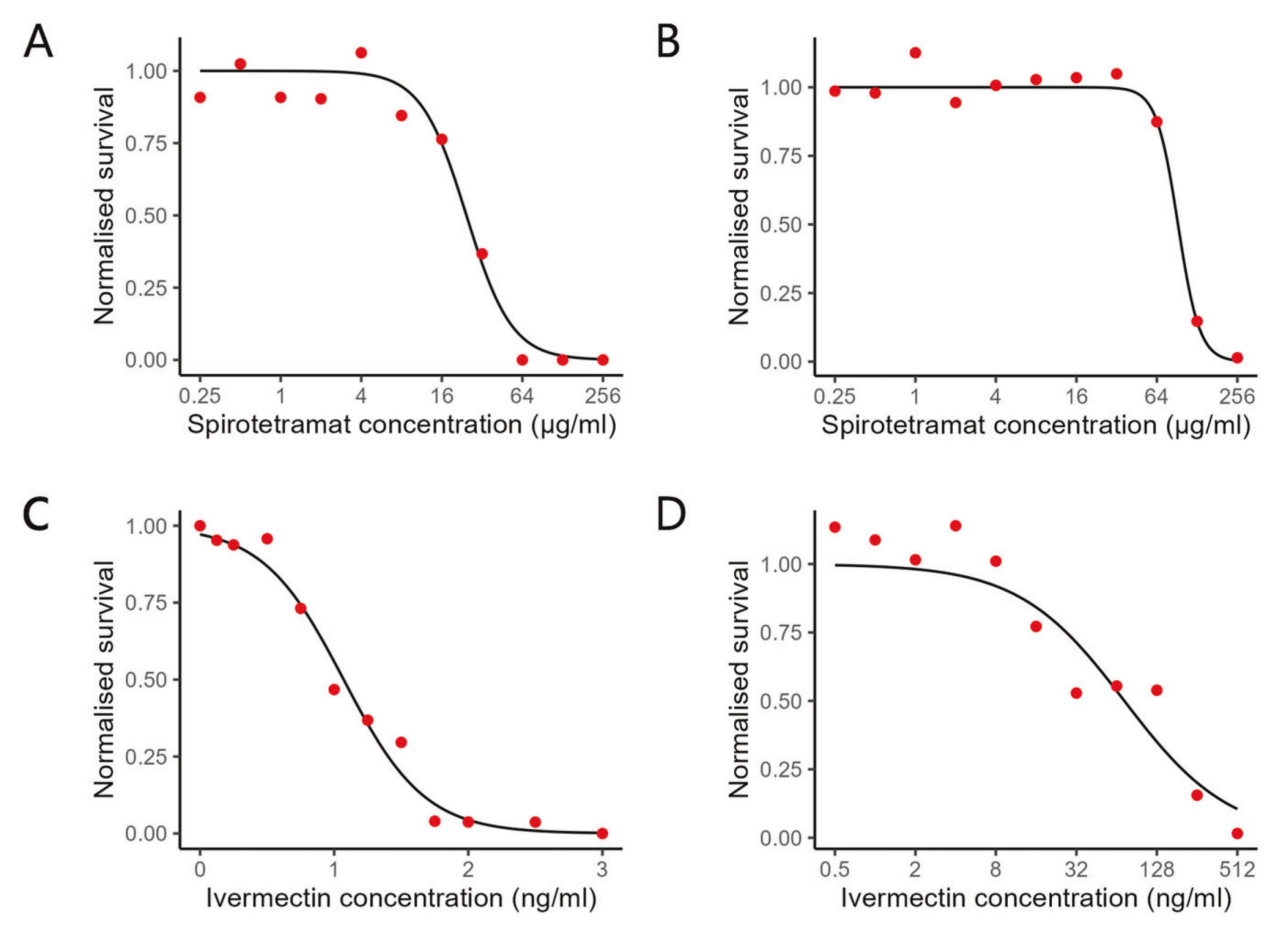
Dose-survival relationship on spirotetramat and ivermectin. 100 synchronised eggs were incubated with compound concentrations ranging from 0.125 to 256 μg/ml for 4 days. Survival was measured as the number of gravid adults on day 4, which was then normalised to survival on vehicle-only control. The mean of 4 repeats is shown. **A** Dose-survival curve of the sensitive PD4792 strain on spirotetramat. **B** Dose-survival curve of the resistant SR42 strain on spirotetramat. **C** Dose-survival curve of the sensitive PD4792 strain on ivermectin. **D** Dose-survival curve of the resistant JD608 strain on ivermectin.

To determine the level of resistance conferred by resistant genotypes, nematode survival data was collected over a wide range of drug concentrations. On spirotetramat, the susceptible strain (PD4792) has an EC_50_ of 24.4 μg/ml (Fig. 2A), and the resistant strain (SR42) has an EC_50_ of 93.3 μg/ml (Fig. 2B), approximately 3.82-fold higher than that of the susceptible strain. On ivermectin, the same susceptible strain (PD4792) has an EC_50_ of 1.07 ng/ml (Fig. 2C), and the resistant strain JD608 has a much increased EC_50_ of 50.1 ng/ml (Fig. 2D).

### Life-history and fitness measurement of relevant *C. elegans* strains

Life-history attributes such as generation time and fecundity underpin fitness of an animal bearing genetic mutations, and forms the foundation upon which selection acts. Discrepancies in life-history traits between competing strains could exert a significant influence on evolution trajectories, even in the absence of selection. Any effect due to the fitness of strains involved in resistance selection will influence the accuracy of the in silico prediction, therefore it was necessary to establish baseline fitness values from experimentation.

**Fig. 3:**
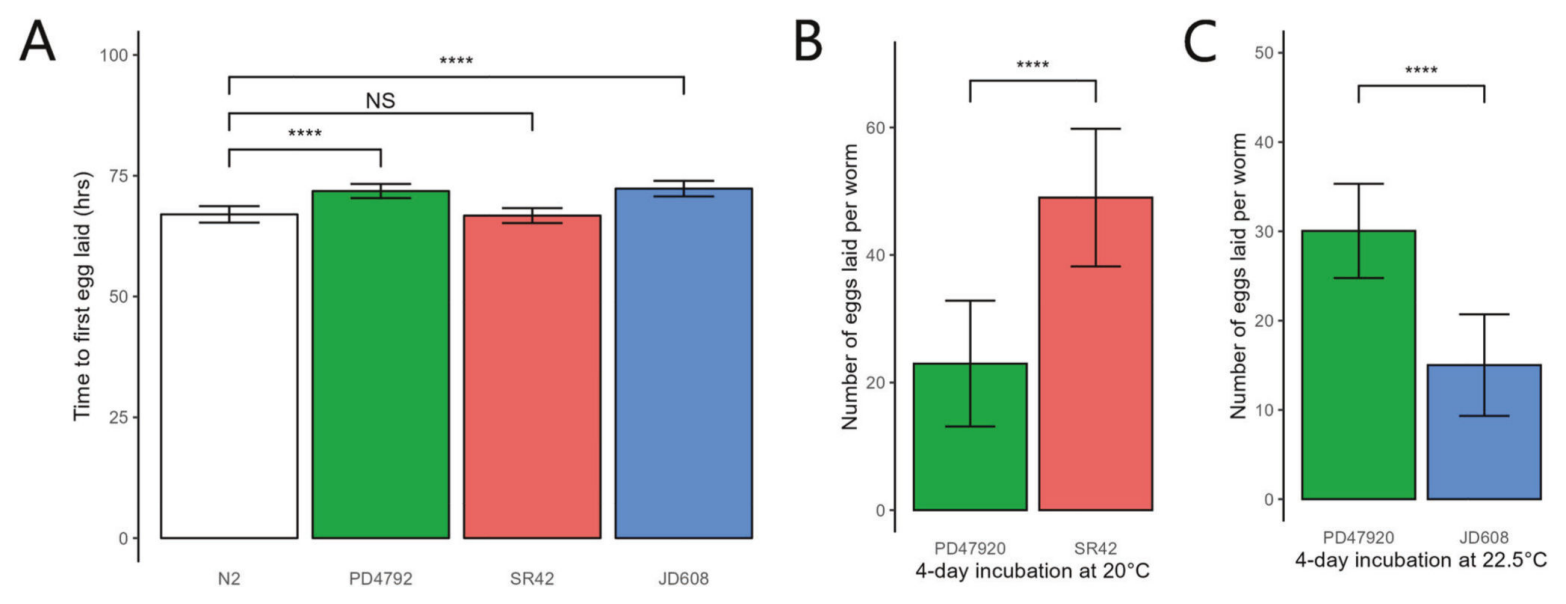
Baseline fitness evaluations of worm strains used for experimental evolution. **A** Developmental timing assay. At hour 0, 50 eggs were seeded on clean NGM plates. Plates were monitored once every hour after hour 64 to check for egg production, the first hour when eggs were seen on the plate was recorded. **** = *p* < 0.0001. NS = not significant, *p* > 0.05. *n* = *12*. Error bars represent SD (standard deviation). **B** 96 h fecundity comparison between PD4792 and SR42, incubated at 20 °C. 1–2 eggs were seeded on each well on day 0. On day 4, the total number of adult worms and laid eggs in each well were counted and the mean number of eggs laid per worm recorded. **** = *p* < 0.0001. *n* = *12*. Error bars represent SD (standard deviation). **C** 96 h fecundity comparison between PD4792 and JD608, incubated at 22.5 °C. **** = *p* < 0.0001. *n* = *12*. Error bars represent SD (standard deviation).

To test this, we investigated egg-to-egg generation time as a measure for the rate of biological development, which could have significant effects on evolutionary outcomes (Rosenheim and Tabashnik 1990). Using time-to-first-egg-production as a proxy, we found that the susceptible PD4792 strain laid its first eggs an average of 4.83 h later than the laboratory reference N2 strain (Fig. 3A, two-sided unpaired Student’s t-test, *p* = 1.915 × 10^−7^), and a similar delay of 5.33 h was observed for the ivermectin-resistant JD608 compared with the same N2 control (Fig. 3A, two-sided unpaired t-test, *p* = 7.788 × 10^−8^). There was no significant difference between the generation time of the control N2 strain and the spirotetramat-resistant strain SR42 (Fig. 3A, two-sided unpaired Student’s t-test, *p* = 0.7103). The delay in JD608 development was likely the result of a direct fitness cost associated with harbouring the triple deletion genotype, as confirmed by a recent study (Shaver et al. 2024). On the other hand, the delay in PD4792 development may be the detrimental effect of harbouring chromosomally integrated GFP transgenes, possibly at high copy numbers, to display a bright fluorescence signal.

The difference in generation time was also reflected in strain fecundity. Fecundity data was collected by counting the number of eggs laid per worm in 12-well plates four days after eggs were initially seeded. With a comparatively faster developmental rate, the spirotetramat-resistant SR42 strain had laid significantly more eggs by the 96 h mark compared to the susceptible PD4792 strain (Fig. 3B, Student’s two-sample unpaired t-test, *p* = 5.688 × 10^−6^). Despite similar developmental timing, the ivermectin-resistant JD608 strain produced fewer eggs compared to the susceptible strain (Fig. 3C, Student’s two-sample unpaired t-test, *p* = 1.75 × 10^−6^). In the subsequent in vivo model, eggs laid after the 96 h mark will not contribute the genetic makeup of the ensuing generation, hence they were not included in the fecundity assay.

### Multi-generation competitive microevolution

Having established the baseline fitness parameters, we conducted multi-generation competitive microevolution assays between the susceptible and resistant strains to probe resistance evolution dynamics during the selection phase. Resistant homozygotic individuals were introduced to a pool of homozygotic susceptible individuals at a set frequency, and the population was then passaged on plates with or without compound for a number of generations. Due to the predominantly selfing mode of *C. elegans* reproduction, no heterozygotes were observed throughout the course of the microevolution assay. In addition, we also did not observe any outcrossing via rarely occurring males in any of the tested conditions.

**Fig. 4:**
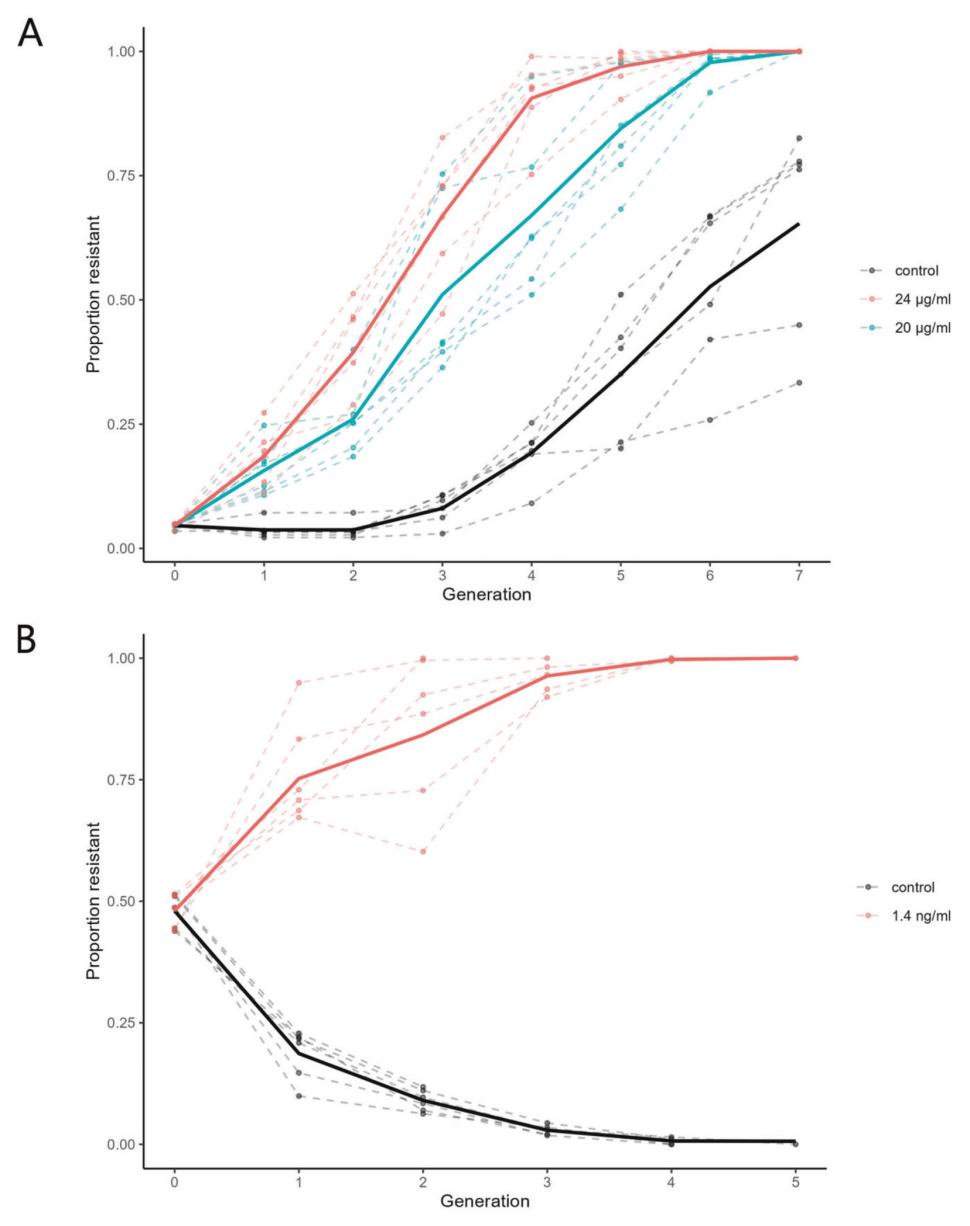
Resistance microevolution on spirotetramat and ivermectin. The proportion of resistant worms was recorded at the end of each generation by counting the number of fluorescent susceptible animals of the total number of animals in a sample. Six biological replicates were conducted for each condition. Dashed lines represent each of the 6 replicates, solid lines represent the replicate means. **A** Experimental evolution on spirotetramat. 5% of homozygotic resistant eggs were mixed with eggs from the susceptible strain. Selection was performed on vehicle control, 20 μg/ml, and 24 μg/ml spirotetramat plates. **B** Experimental evolution on ivermectin. 50% of homozygotic resistant eggs were mixed with eggs from the susceptible strain. Selection was performed on vehicle control and 1.4 ng/ml ivermectin plates.

For spirotetramat, a moderate selection strength corresponding to ~50% survival of the susceptible individuals were chosen. Specifically, three experimental conditions were tested: 20 μg/ml, 24 μg/ml, and solvent-only control, starting with a frequency of 5% resistance in every lineage. Both treatment conditions displayed clear resistance selection from generation 1 onwards, and resulted in much higher resistance allele frequency compared to the control condition throughout (Fig. 4A). Furthermore, the experimental assay was able to distinguish between the two doses of spirotetramat, which differed by only a small amount of selection strength. The increased fecundity of the spirotetramat-resistant strain (Fig. 3B) was most clearly observed in the solvent-only control, where despite the lack of selection the frequency of resistant individuals increased, albeit seemingly with a two-generation delay. These results are in accordance with single-generation dose-response and fitness data (Figs. 2A, B, 3A, B), and show that the multi-generation competition assay does capture essential characteristics of the expected evolutionary trajectories.

For ivermectin, a high selection strength corresponding to ~10% survival of the susceptible individuals were chosen. Two conditions were tested: 1.4 ng/ml and solvent-only control. Resistance was introduced to a susceptible population at 50% abundance to account for the fitness defect of the ivermectin-resistant JD608 strain (Fig. 3C). We expected that under ivermectin selection, the JD608 genotype would overcome its natural disadvantage and fix itself in the population, whereas under no selection, this genotype would be purged from the population due to its fitness defect. The experimental results confirmed these predictions (Fig. 4B). With selection at 1.4 ng/ml, fixation happened as early as generation 2 among the 6 biological replicate lineages. In one lineage, resistance frequency decreased despite selection, likely demonstrating the significant cost associated with the JD608 genetic background. Without selection, the ivermectin-resistant genotype was rapidly purged from the population and went almost extinct in all replicate lines by generation 4.

### Predicting microevolution trajectories with a computational population genetics model

**Fig. 5:**
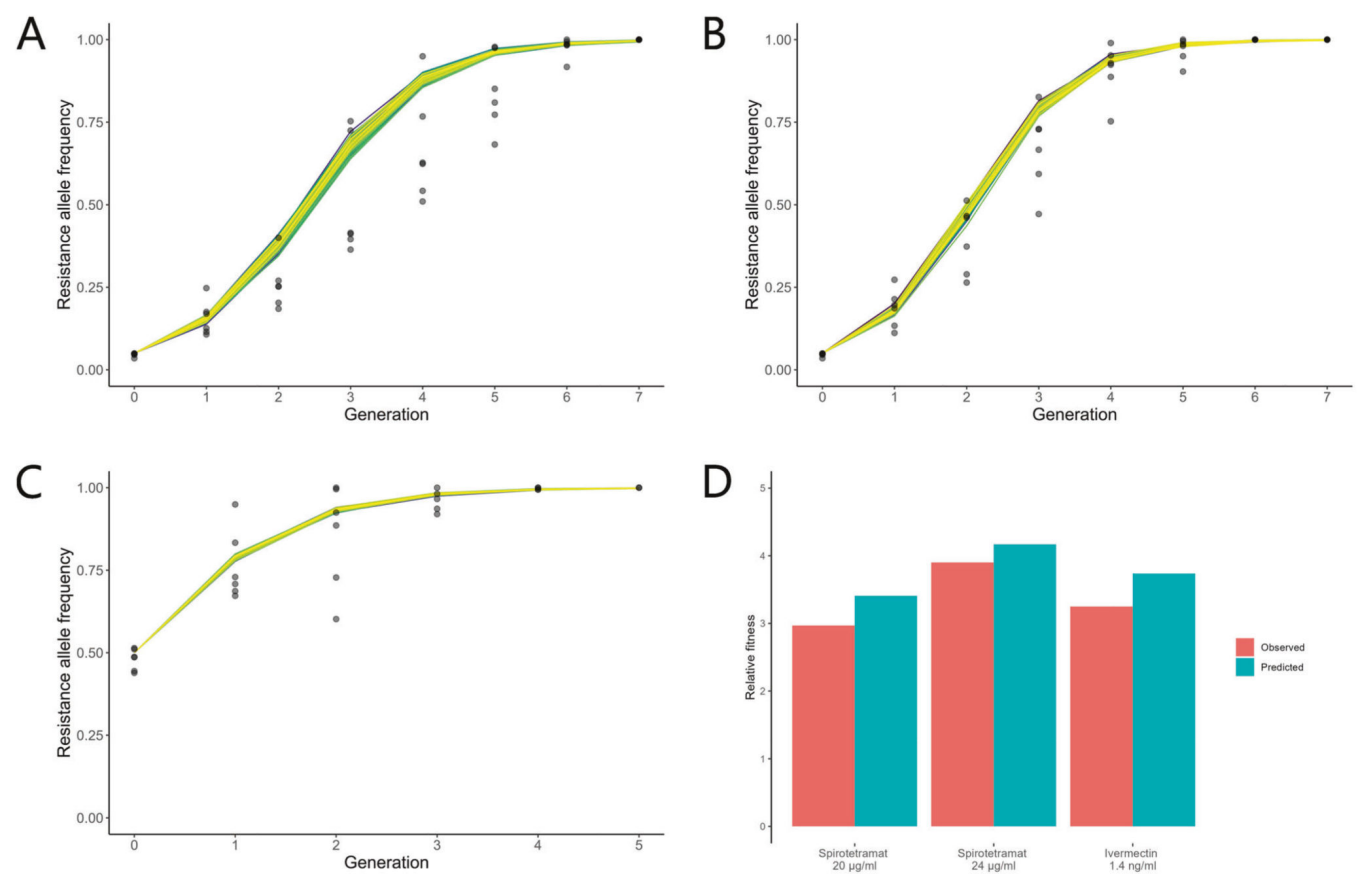
Concordance between in silico and in vivo models. The in silico model utilised experimental data from single-generation fecundity and compound survival assays to predict multi-generation resistance microevolution under selection. One hundred predictions involving stochasticity were generated for each condition. In silico model predictions are shown in colour, overlayed with in vivo data in black. **A** Spirotetramat selection at 20 μg/ml. **B** Spirotetramat selection at 24 μg/ml. **C** Ivermectin selection at 1.4 ng/ml. **D** Comparison of in vivo and in silico predictions of relative fitness of the resistance homozygote over the susceptible homozygote $\left(\frac{W_{AA}}{W_{aa}}\right)$.

An in silico population genetics model was constructed following Wright-Fisher dynamics (Fisher 1930; Wright 1931) to replicate in vivo resistance microevolution, incorporating stochasticity by sampling from a binomial distribution (for full in silico model code see S3). The in silico model assumes that the population is homogenous and generations are discrete and non-overlapping, as was the case for the in vivo experiments. To parameterise the in silico model, the fitness of the genotypes involved in resistance selection were calculated from the single-generation experimental data (Figs. 2, 3), specifically as the product of the baseline fecundity and the percentage survival on given compound concentration as observed from dose-survival data. The in silico model thus parameterised would be expected to predict the fraction of resistance individuals in the population at the end of each generation. Overall, the output of the stochastic simulation based on single-generation experimental data was found to closely resemble evolutionary trajectories of the multi-generation microevolution in all three compound selection conditions (Fig. 5A–C). Experimental data presented with higher noise than simulation predictions. One possibility is that fluctuations in effective population size could contribute to the increased variance; this is possible because although the population size was kept constant at 7500 in each generation, not all worms may have survived to adulthood. In order to test for the contribution of population size to variance, we ran simulations with much lower population sizes and found that recapitulation of the experimental variance was only achieved when the population size was dropped to 100. Thus we can conclude that the effective population size was unlikely to be a major source of variance in the experiment.

A quantitative comparison of in vivo data and in silico predictions was established through comparing the relative fitness of the resistant strain against the susceptible strain in each condition (Fig. 5D). The in silico model explicitly records the fitness of the resistance and susceptible strains, hence a direct calculation yields the relative fitness. From in vivo data, relative fitness was calculated through fitting a linear model whose gradient reflected the selection coefficient (see S4), which was then converted to relative fitness (see Materials and Methods). In general, the in silico model captured the characteristics of the biological system under investigation, and was an accurate predictor of multi-generation microevolution dynamics within the current framework.

## Discussion

In this work, we used a simple *C. elegans* model to demonstrate the feasibility of using nematodes as a model organism for understanding arthropod pesticide resistance, combining experimental and theoretical insights. The ease of maintaining nematodes in controlled laboratory conditions at large population sizes make them a particularly suitable model for the purpose of dissecting pesticide resistance evolution in a controlled laboratory environment. Having established that wild *C. elegans* isolates can evolve resistance to pesticidal chemicals, we used previously characterised resistance alleles in lab-adapted nematode strains to probe whether an appropriately constructed in silico model can predict in vivo multi-generation microevolution trajectories. Comparing the results of the in vivo and in silico models, we show that indeed such prediction is possible at a reasonable accuracy.

There is significant value in creating a hybrid experimental-theoretical model for dissecting resistance evolution dynamics. The possibility of accurate multi-generation resistance evolution prediction from baseline data is appealing, not only because it lends support to the validity of commonly used theoretical frameworks, also because it creates bidirectional synergy. On one hand, it allows scenarios that are difficult or impossible to test in an experimental setting to be explored computationally, given experimentally-determined starting points. On the other hand, with the extensive available literature on theoretical and computational modelling of pesticide resistance evolution (Comins 1986; Roush 1989; Tabashnik 1989; REX Consortium 2013; Helps et al. 2017; Levick et al. 2017; Barbosa et al. 2018; Helps et al. 2020; South et al. 2020; Madgwick and Kanitz 2022b; Hobbs et al. 2023), there is much to be gained from experimental studies that deliberately test the hypotheses identified in in silico work, and provide empirical evidence for their validity.

As proof-of-concept, this study adopted a simplified experimental setup in order to establish a foundation to bridge experimental and theoretical insights. Neither the in vivo nor the in silico model captured the full range of complexities that are likely to be involved in a field-realistic resistance evolution scenario. Two examples of such simplifications and the associated limitations will be examined here. First is the use of discrete, non-overlapping generations in both models. In the field, insect populations consist of continuous generations with complex population age structures (Bluher et al. 2020; Cook et al. 2024). This is especially problematic if the pesticidal compound in question targets specific developmental stages of insect development (Rodriguez-Saona et al. 2016). Theoretical resistance modelling work has explored the impact of population structure on resistance evolution outcomes (Sudo et al. 2017). Additionally, it is possible to convert between discrete-time and continuous-time resistance evolution dynamics through continuous stochastic stimulations or analytical work, an example of which can be seen in Magdwick and Kanitz 2022a (Madgwick and Kanitz 2022a).

Another example where the current in vivo and in silico models simplify from field-realistic evolutionary scenarios is their disregard for changes in population density. In both the in vivo and in silico models, the population density was kept constant by starting every generation with the same population size to mitigate any density-dependent effects. In reality, as pesticidal compounds are applied to a pest population, it necessarily follows that the total insect population size must shrink. Should the control be effective, such decrease would be dramatic, and result in a minute fraction of the initial population surviving compound treatment. From existing work in dioecious animals, it is known that the relationship between mating success and population density is complex and can lead to unexpected effects (Myhre et al. 2017; Watts et al. 2022). Additionally, altering population size alone can influence resistance evolution dynamics. Stochastic effects, i.e., genetic drift, become more dominant with low population sizes which could lead to highly varying outcomes.

The study reported here focuses on the effects of selection upon existing standing variation, and does not explore the impact of de novo mutations. In every replicate where selection was imposed, the resistant strain completely displaced the susceptible strain within a small number of generations. Since fixation of the known resistance-conferring allele occurred in every biological replicate where selection had been imposed, it suggests that high-level de novo resistance mutations are sufficiently rare, and not likely to arise within the experimental timeframe above from the background mutation rate of 10^−8^ to 10^−9^ per nucleotide per generation (Denver et al. 2004). With this in mind, it would be possible to alter the background mutation rate by mutagenesis, a well-established procedure in *C. elegans*, and adapt the current system to explore the dynamics of resistance emergence of which little is currently known. Imposing selection on a mutagenised population could generate a wealth of insight on the genetic features promoting resistance evolution, such as resistance driven by multiple loci as well as those that carry fitness trade-offs. The landscape of these genetic features could reveal information about how insecticide resistance emerges in the field.

Beyond target-site mutations, another intriguing possibility lies in gene expression changes. It is well-known that the upregulation of detoxification enzymes can lead to significant increases in xenobiotic resistance in insects (Amezian et al. 2021). The relationship between resistance driven by gene expression changes and those driven by mutations in the compound target site is not well understood. The current system we have established using *C. elegans* is well-suited for addressing the role played by non-target site mutations in resistance evolution.

With the hybrid experimental-theoretical system we have developed, further questions within the field of pesticide resistance evolution can be addressed. Work is ongoing to translate the system to a dioecious mating strain of *C. elegans*, using strains harbouring *fog-2* loss-of-function mutations (Hu et al. 2019). The dioecious system will better resemble resistance evolution dynamics in the vast majority of pest insect species, and be well-suited to address the question of choosing an optimal strategy for resistance management. Theoretical work and computational simulations have provided guideline and evidence in favour of applying multiple insecticidal compounds concurrently to limit resistance spread (Comins 1986; Roush 1989; Tabashnik 1989; Levick et al. 2017; South and Hastings 2018; Helps et al. 2020; Madgwick and Kanitz 2022a, 2024). However, as of yet it has not been possible to verify these insights in an in vivo system. The dioecious system under development will be well-suited for exploring the evolutionary dynamics of resistant management strategies involving the use of two compounds in combination.

Overall, the present study establishes a proof-of-concept model for understanding and predicting insecticide resistance evolution in the model organism *C. elegans*. While not an insect, this nematode model shows promise due to conservation of evolutionary dynamics and biochemical pathways, as well as experimentally scalability and tractability. Using *C. elegans* with known genetic resistance, we showed that an appropriately constructed in silico model can predict microevolution dynamics under a simplified laboratory setting. *C. elegans* can be an effective model for understanding the evolutionary consequences of the rapid selection imposed by insecticides, and validate current and future computational frameworks. With iterations and extensions, the experimental-theoretical paradigm established here will add to the existing toolbox aiding the prediction of pesticide resistance evolution in the field.

## Supplementary Material

**Supplementary information** The online version contains supplementary material available at https://doi.org/10.1038/s41437-025-00781-x.

Supplementary materials

## Figures and Tables

**Table 1:** Criteria of evaluation for chemical susceptibility screen.

	Number of survived worms	Defects		
		Development	Movement	Reproduction
1 (most susceptible)	0	No survival	No survival	No survival
2	1−5	Under-developed worms observed	Paralysed or severe movement defect	No or very few eggs/offspring visible
3	5−7	Limited or no observable developmental defect	Some movement defect	Some offspring and eggs visible
4 (least susceptible)	8−10	No developmental defect	No movement defect	Lots of eggs and offspring visible

## Data Availability

All original data relevant to the study has been archived on Dryad (https://doi.org/10.5061/dryad.d7wm37qd1). The in silico model code is available as S3 in the Supplementary Materials.

## References

[R1] Amezian D, Nauen R, Le Goff G (2021). Transcriptional regulation of xenobiotic detoxification genes in insects - An overview. Pestic Biochem Physiol.

[R2] Ashburner MG, Kent G, Hawley RS (2005). Drosophila: A Laboratory Handbook.

[R3] Ashburner MTJN (1978). The laboratory culture of Drosophila.

[R4] Ballu A, Deredec A, Walker AS, Carpentier F (2021). Are Efficient-Dose Mixtures a Solution to Reduce Fungicide Load and Delay Evolution of Resistance? An Experimental Evolutionary Approach. Microorganisms.

[R5] Barbosa S, Kay K, Chitnis N, Hastings IM (2018). Modelling the impact of insecticide-based control interventions on the evolution of insecticide resistance and disease transmission. Parasit Vectors.

[R6] Bass C, Denholm I, Williamson MS, Nauen R (2015). The global status of insect resistance to neonicotinoid insecticides. Pestic Biochem Physiol.

[R7] Bian T, Zhu X, Guo J, Zhuang Z, Cai Z, Zhao X (2018). Toxic effect of the novel chiral insecticide IPP and its biodegradation intermediate in nematode Caenorhabditis elegans. Ecotoxicol Environ Saf.

[R8] Bluher SE, Miller SE, Sheehan MJ (2020). Fine-Scale Population Structure but Limited Genetic Differentiation in a Cooperatively Breeding Paper Wasp. Genome Biol Evol.

[R9] Braendle C, Paaby A (2024). Life history in Caenorhabditis elegans: from molecular genetics to evolutionary ecology. Genetics.

[R10] Casida JE, Durkin KA (2017). Pesticide Chemical Research in Toxicology: Lessons from Nature. Chem Res Toxicol.

[R11] Catania F, Kauer MO, Daborn PJ, Yen JL, Ffrench-Constant RH, Schlotterer C (2004). World-wide survey of an Accord insertion and its association with DDT resistance in Drosophila melanogaster. Mol Ecol.

[R12] Comins HN (1977). The management of pesticide resistance. J Theor Biol.

[R13] Comins HN (1986). Tactics for resistance management using multiple pesticides. Agriculture, Ecosyst Environ.

[R14] Cook PA, Costello RA, Brodie Iii ED, Formica V (2024). Population age structure shapes selection on social behaviour in a long-lived insect. Philos Trans R Soc Lond B Biol Sci.

[R15] Curtis CF (1985). Theoretical models of the use of insecticide mixtures for the management of resistance. Bulletin Entomological Res.

[R16] Dennis EJ, Dobosiewicz M, Jin X, Duvall LB, Hartman PS, Bargmann CI, Vosshall LB (2018). A natural variant and engineered mutation in a GPCR promote DEET resistance in C. elegans. Nature.

[R17] Dent JA, Smith MM, Vassilatis DK, Avery L (2000). The genetics of ivermectin resistance in Caenorhabditis elegans. Proc Natl Acad Sci USA.

[R18] Denver DR, Morris K, Lynch M, Thomas WK (2004). High mutation rate and predominance of insertions in the Caenorhabditis elegans nuclear genome. Nature.

[R19] Dickinson DJ, Goldstein B (2016). CRISPR-Based Methods for Caenorhabditis elegans Genome Engineering. Genetics.

[R20] Fisher RA (1930). The genetic theory of natural selection.

[R21] Frezal L, Felix MA (2015). C. elegans outside the Petri dish. Elife.

[R22] Furnival-Adams J, Kiuru C, Sagna AB, Mouline K, Maia M, Chaccour C (2024). Ivermectin resistance mechanisms in ectoparasites: a scoping review. Parasitol Res.

[R23] Gaines TA, Duke SO, Morran S, Rigon CAG, Tranel PJ, Kupper A, Dayan FE (2020). Mechanisms of evolved herbicide resistance. J Biol Chem.

[R24] Georghiou G (1972). The Evolution of Resistance to Pesticides. Annual Rev Ecol Syst.

[R25] Ghosh R, Andersen EC, Shapiro JA, Gerke JP, Kruglyak L (2012). Natural variation in a chloride channel subunit confers avermectin resistance in C. elegans. Science.

[R26] Gould F, Brown ZS, Kuzma J (2018). Wicked evolution: Can we address the socio-biological dilemma of pesticide resistance?. Science.

[R27] Guest M, Kriek N, Flemming AJ (2020). Studies of an insecticidal inhibitor of acetyl-CoA carboxylase in the nematode *C. elegans*. Pestic Biochem Physiol.

[R28] Gutbrod P, Gutbrod K, Nauen R, Elashry A, Siddique S, Benting J (2020). Inhibition of acetyl-CoA carboxylase by spirotetramat causes growth arrest and lipid depletion in nematodes. Sci Rep.

[R29] Hahnel SR, Zdraljevic S, Rodriguez BC, Zhao Y, McGrath PT, Andersen EC (2018). Extreme allelic heterogeneity at a Caenorhabditis elegans beta-tubulin locus explains natural resistance to benzimidazoles. PLoS Pathog.

[R30] Haldane JB (1927). A Mathematical Theory of Natural and Artificial Selection, Part V: Selection and Mutation. Mathematical Proc Camb Philos Soc.

[R31] Hawkins NJ, Bass C, Dixon A, Neve P (2019). The evolutionary origins of pesticide resistance. Biol Rev Camb Philos Soc.

[R32] Helps JC, Paveley ND, van den Bosch F (2017). Identifying circumstances under which high insecticide dose increases or decreases resistance selection. J Theor Biol.

[R33] Helps JC, Paveley ND, White S, van den Bosch F (2020). Determinants of optimal insecticide resistance management strategies. J Theor Biol.

[R34] Hobbelen PH, Paveley ND, van den Bosch F (2014). The emergence of resistance to fungicides. PLoS One.

[R35] Hobbs NP, Weetman D, Hastings IM (2023). Insecticide resistance management strategies for public health control of mosquitoes exhibiting polygenic resistance: A comparison of sequences, rotations, and mixtures. Evol Appl.

[R36] Hu S, Skelly LE, Kaymak E, Freeberg L, Lo TW, Kuersten S (2019). Multi-modal regulation of C. elegans hermaphrodite spermatogenesis by the GLD-1-FOG-2 complex. Dev Biol.

[R37] Jacomb F, Marsh J, Holman L (2016). Sexual selection expedites the evolution of pesticide resistance. Evolution.

[R38] Johnston WL, Dennis JW (2012). The eggshell in the *C. elegans* oocyte-to-embryo transition. Genesis.

[R39] Kaduskar B, Kushwah RBS, Auradkar A, Guichard A, Li M, Bennett JB (2022). Reversing insecticide resistance with allelic-drive in Drosophila melanogaster. Nat Commun.

[R40] Kim HM, Colaiacovo MP (2019). CRISPR-Cas9-Guided Genome Engineering in Caenorhabditis elegans. Curr Protoc Mol Biol.

[R41] Kimura M (1962). On the probability of fixation of mutant genes in a population. Genetics.

[R42] Lagator M, Vogwill T, Colegrave N, Neve P (2013). Herbicide cycling has diverse effects on evolution of resistance in Chlamydomonas reinhardtii. Evol Appl.

[R43] Laing R, Gillan V, Devaney E (2017). Ivermectin - Old Drug, New Tricks?. Trends Parasitol.

[R44] Le Goff G, Hilliou F (2017). Resistance evolution in Drosophila: the case of CYP6G1. Pest Manag Sci.

[R45] Levick B, South A, Hastings IM (2017). A Two-Locus Model of the Evolution of Insecticide Resistance to Inform and Optimise Public Health Insecticide Deployment Strategies. PLoS Comput Biol.

[R46] Lopes PC, Sucena E, Santos ME, Magalhaes S (2008). Rapid experimental evolution of pesticide resistance in C. elegans entails no costs and affects the mating system. PLoS One.

[R47] Lucas JA, Hawkins NJ, Fraaije BA (2015). The evolution of fungicide resistance. Adv Appl Microbiol.

[R48] Lueke B, Douris V, Hopkinson JE, Maiwald F, Hertlein G, Papapostolou KM (2020). Identification and functional characterization of a novel acetyl-CoA carboxylase mutation associated with ketoenol resistance in Bemisia tabaci. Pestic Biochem Physiol.

[R49] Madgwick PG, Kanitz R (2022a). Beyond redundant kill: A fundamental explanation of how insecticide mixtures work for resistance management. Pest Manag Sci.

[R50] Madgwick PG, Kanitz R (2022b). Modelling new insecticide-treated bed nets for malaria-vector control: how to strategically manage resistance?. Malar J.

[R51] Madgwick PG, Kanitz R (2024). What is the value of rotations to insecticide resistance management?. Pest Manag Sci.

[R52] Madgwick PG, Tunstall T, Kanitz R (2024). Evolutionary rescue in resistance to pesticides. Proc Biol Sci.

[R53] Mani GS (1985). Evolution of resistance in the presence of two insecticides. Genetics.

[R54] Martin F, Svansson V, Eydal M, Oddsdottir C, Ernback M, Persson I, Tyden E (2021). First Report of Resistance to Ivermectin in Parascaris univalens in Iceland. J Parasitol.

[R55] Mateos Fernandez R, Petek M, Gerasymenko I, Jutersek M, Baebler S, Kallam K (2022). Insect pest management in the age of synthetic biology. Plant Biotechnol J.

[R56] May, RD, AP (1986). Pesticide resistance: strategies and tactics for management.

[R57] McKenzie JA, Batterham P (1998). Predicting insecticide resistance: mutagenesis, selection and response. Philos Trans R Soc Lond B Biol Sci.

[R58] Muschiol D, Schroeder F, Traunspurger W (2009). Life cycle and population growth rate of Caenorhabditis elegans studied by a new method. BMC Ecol.

[R59] Myhre AM, Engen S, BE SA (2017). Effective size of density-dependent two-sex populations: the effect of mating systems. J Evol Biol.

[R60] Neve P, Powles S (2005). Recurrent selection with reduced herbicide rates results in the rapid evolution of herbicide resistance in Lolium rigidum. Theor Appl Genet.

[R61] Omrane S, Sghyer H, Audeon C, Lanen C, Duplaix C, Walker AS, Fillinger S (2015). Fungicide efflux and the MgMFS1 transporter contribute to the multidrug resistance phenotype in Zymoseptoria tritici field isolates. Environ Microbiol.

[R62] Parker WE, Howard JJ, Foster SP, Denholm I (2006). The effect of insecticide application sequences on the control and insecticide resistance status of the peachpotato aphid, Myzus persicae (Hemiptera:Aphididae), on field crops of potato. Pest Manag Sci.

[R63] Pertrungaro GMYBT (2021). Antibiotic resistance: Insights from evolution experiments and mathematical modeling. Curr Opin Syst Biol.

[R64] Porta-de-la-Riva M, Fontrodona L, Villanueva A, Ceron J (2012). Basic Caenorhabditis elegans methods: synchronization and observation. J Vis Exp.

[R65] Prabhaker N, Toscano NC, Henneberry TJ (1998). Evaluation of Insecticide Rotations and Mixtures as Resistance Management Strategies for Bemisia argentifolii (Homoptera: Aleyrodidae). Journal Economic Entomol.

[R66] Qian H, Robertson AP, Powell-Coffman JA, Martin RJ (2008). Levamisole resistance resolved at the single-channel level in Caenorhabditis elegans. FASEB J.

[R67] REX-Consortium (2013). Heterogeneity of selection and the evolution of resistance. Trends Ecol Evol.

[R68] Reynolds A, Lindstrom J, Johnson PC, Mable BK (2016). Evolution of drug-tolerant nematode populations in response to density reduction. Evol Appl.

[R69] Rodriguez-Saona C, Wanumen AC, Salamanca J, Holdcraft R, Kyryczenko-Roth V (2016). Toxicity of Insecticides on Various Life Stages of Two Tortricid Pests of Cranberries and on a Non-Target Predator. Insects.

[R70] Rosenheim J, Tabashnik BE (1990). Evolution of pesticide resistance: interactions between generation time and genetic, ecological, and operational factors. J Econ Entomol.

[R71] Roush RT (1989). Designing resistance management programs: How can you choose?. Pesticide Sci.

[R72] Roux F, Camilleri C, Berard A, Reboud X (2005). Multigenerational versus single generation studies to estimate herbicide resistance fitness cost in Arabidopsis thaliana. Evolution.

[R73] Rufener L, Bedoni N, Baur R, Rey S, Glauser DA, Bouvier J (2013). acr-23 Encodes a monepantel-sensitive channel in Caenorhabditis elegans. PLoS Pathog.

[R74] Sadia CG, Bonneville JM, Zoh MG, Fodjo BK, Kouadio FA, Oyou SK (2024). The impact of agrochemical pollutant mixtures on the selection of insecticide resistance in the malaria vector Anopheles gambiae: insights from experimental evolution and transcriptomics. Malar J.

[R75] Sammons RD, Gaines TA (2014). Glyphosate resistance: state of knowledge. Pest Manag Sci.

[R76] Schedl T, Kimble J (1988). fog-2, a germ-line-specific sex determination gene required for hermaphrodite spermatogenesis in Caenorhabditis elegans. Genetics.

[R77] Schleker ASS, Rist M, Matera C, Damijonaitis A, Collienne U, Matsuoka K (2022). Mode of action of fluopyram in plant-parasitic nematodes. Sci Rep.

[R78] Schulenburg H, Felix MA (2017). The Natural Biotic Environment of Caenorhabditis elegans. Genetics.

[R79] Seong KM, Mittapalli O, Clark JM, Pittendrigh BR (2019). A review of DDT resistance as it pertains to the 91-C and 91-R strains in Drosophila melanogaster. Pestic Biochem Physiol.

[R80] Shaver AO, Miller IR, Schaye ES, Moya ND, Collins JB, Wit J (2024). Quantifying the fitness effects of resistance alleles with and without anthelmintic selection pressure using Caenorhabditis elegans. PLoS Pathog.

[R81] Shyma KP, Gupta JP, Parsani HR, Ankuya KJ, Singh V (2021). Ivermectin resistance in the multi-host tick Hyalomma anatolicum (Acari: Ixodidae) in India. Ticks Tick Borne Dis.

[R82] Siddiqui JA, Fan R, Naz H, Bamisile BS, Hafeez M, Ghani MI (2022). Insights into insecticide-resistance mechanisms in invasive species: Challenges and control strategies. Front Physiol.

[R83] Sluder A, Shah S, Cassayre J, Clover R, Maienfisch P, Molleyres LP (2012). Spiroindolines identify the vesicular acetylcholine transporter as a novel target for insecticide action. PLoS One.

[R84] South A, Hastings IM (2018). Insecticide resistance evolution with mixtures and sequences: a model-based explanation. Malar J.

[R85] South A, Lees R, Garrod G, Carson J, Malone D, Hastings I (2020). The role of windows of selection and windows of dominance in the evolution of insecticide resistance in human disease vectors. Evol Appl.

[R86] Stiernagle T (2006). Maintenance of *C. elegans*.

[R87] Sudo M, Takahashi D, Andow DA, Suzuki Y, Yamanaka T (2017). Optimal management strategy of insecticide resistance under various insect life histories: Heterogeneous timing of selection and interpatch dispersal. Evol Appl.

[R88] Tabashnik BE (1989). Managing resistance with multiple pesticide tactics: theory, evidence, and recommendations. J Econ Entomol.

[R89] Troczka B, Zimmer CT, Elias J, Schorn C, Bass C, Davies TG (2012). Resistance to diamide insecticides in diamondback moth, Plutella xylostella (Lepidoptera: Plutellidae) is associated with a mutation in the membrane-spanning domain of the ryanodine receptor. Insect Biochem Mol Biol.

[R90] Watson GB, Chouinard SW, Cook KR, Geng C, Gifford JM, Gustafson GD (2010). A spinosyn-sensitive Drosophila melanogaster nicotinic acetylcholine receptor identified through chemically induced target site resistance, resistance gene identification, and heterologous expression. Insect Biochem Mol Biol.

[R91] Watts JC, Hebets EA, Tenhumberg B (2022). Mate Sampling Behavior Determines the Density Dependence of Sexual Selection. Am Nat.

[R92] Welz C, Kruger N, Schniederjans M, Miltsch SM, Krucken J, Guest M (2011). SLO-1-channels of parasitic nematodes reconstitute locomotor behaviour and emo-depside sensitivity in Caenorhabditis elegans slo-1 loss of function mutants. PLoS Pathog.

[R93] Wright S (1931). Evolution in Mendelian Populations. Genetics.

[R94] Yin Y, Miao J, Shao W, Liu X, Zhao Y, Ma Z (2023). Fungicide Resistance: Progress in Understanding Mechanism, Monitoring, and Management. Phytopathology.

[R95] Zhu L, Zhang S, Lu F, Zhang K, Han Q, Ying Q (2021). Cross-resistance, fitness costs, and biochemical mechanism of laboratory-selected resistance to ten-vermectin A in Plutella xylostella. Pest Manag Sci.

[R96] Zoh MG, Bonneville JM, Tutagata J, Laporte F, Fodjo BK, Mouhamadou CS (2021). Experimental evolution supports the potential of neonicotinoid-pyrethroid combination for managing insecticide resistance in malaria vectors. Sci Rep.

