# Supplementary material for: A proof-of-concept experimental-theoretical model to predict pesticide resistance evolution
Source: Heredity (Edinb). Author manuscript; Available in PMC 2026 Apr 22. (PMC7619028; doi:10.1038/s41437-025-00781-x)

**Supplementary Materials**

**S1. Strain name and geographical origins of wild C. elegans isolates used in chemical susceptibility screen**

| Strain | Geographical origin |
| --- | --- |
| N2 | Sampled from Bristol, UK (laboratory-adapted) |
| AB1 | Sampled from Adelaide, Australia |
| CB4851 | Sampled from Bergerac, France |
| CB4853 | Sampled from Altadena, USA |
| CB4854 | Sampled from Altadena, USA |
| CB4856 | Sampled from Hawaii, USA |
| CB4857 | Sampled from Claremont, USA |
| CB4858 | Sampled from Pasadena, CA, USA |
| CX11262 | Sampled from Huntington Gardens, Los Angeles, USA |
| CX11307 | Sampled from Huntington Gardens, Los Angeles, USA |
| ED3005 | Sampled from Edinburgh, Scotland |
| ED3012 | Sampled from Edinburgh, Scotland |
| ED3017 | Sampled from Edinburgh, Scotland |
| JU1088 | Sampled from Kakegawa, Japan |
| JU1400 | Sampled from Sevilla, Spain |
| JU1491 | Sampled from Le Blanc, France |
| JU1580 | Sampled from Orsay, France |
| JU1652 | Sampled from Montevideo, Uruguay |
| JU258 | Sampled from Ribeiro Frio, Madeira |
| JU311 | Sampled from Merlet, France |
| JU363 | Sampled from Franconville, France |
| JU775 | Sampled from Lisbon, Portugal |
| JU792 | Sampled from Frechendets, France |
| LKC34 | Sampled from Madagascar |
| MY23 | Sampled from Münster, NW Germany |
| PB306 | Sampled from Connecticut Valley, USA |
| QX1211 | Sampled from San Francisco, California |

**S2. Compound concentrations used in chemical susceptibility screen of wild *C. elegans* isolates.**

|  | Concentration used in laboratory screen | | Concentration applied in the field |
| --- | --- | --- | --- |
| Compound | **ppm** | **µM** | **ppm** |
| Abamectin | 50 | 55 | 20 |
| Aldicarb | 50 | 253 | NA |
| Amitraz | 50 | 164 | 200 |
| Carbendazim | 50 | 252 | 500 |
| Cartap | 50 | 203 | 500 |
| Cyanotropane | 50 | 697 | NA |
| Cyprodinil | 50 | 214 | 500 |
| DDT | 50 | 136 | NA |
| Diafenthiuron | 5 | 13 | 500 |
| Dieldrin | 50 | 126 | NA |
| Diflubenzuron | 50 | 155 | 65 |
| Fenoxycarb | 50 | 160 | 50 |
| Fenvalerate | 50 | 115 | 200 |
| Fluazinam | 5 | 10 | 500 |
| Flufenoxuron | 50 | 99 | 50 |
| Fluopyram | 5 | 12 | 415 |
| Indoxacarb | 50 | 91 | 150 |
| Levamisole | 50 | 236 | 150 |
| Lufenuron | 50 | 94 | 100 |
| Metaflumizone | 50 | 95 | NA |
| Monepantel | 5 | 10 | 25 |
| Oxamyl | 50 | 220 | 500 |
| Pirimicarb | 50 | 202 | 500 |
| Pyriproxyfen | 50 | 150 | 100 |
| Rotenone | 5 | 12 | 50 |
| Ryanodine | 50 | 98 | NA |
| Spinosad | 50 | 33 | 115 |
| Spiroindoline | 50 | 167 | NA |
| Spirotetramat | 50 | 129 | 500 |

**S3. *In silico* model code in R.**

| library(viridis)  library(reshape2)  library(readxl)  library(ggplot2)  # Model setup  # Constant population size of 7500  # Binomial stochasticity in offspring genotype generation  # Resistance allele present from start at set frequencies  # C. elegans genetics with fully selfing hermaphrodites  # Non-overlapping generations  # Homogeneous population  # No de novo mutation  # MANUAL CHOICE: choose one of three setups  setup = "spirotetramat_moderate"  setup = "spirotetramat_high"  setup = "ivermectin_treatment"  ## The script from this point on can be run without any manual input/changes  # baseline fecundity  fecundity = read_excel("0039 fecundity.xlsx")  fecundity = colMeans(fecundity, na.rm=T)  # baseline survival on compound  load("iv_aa_model.Rdata")  a = log2(1.4) # 1.4ng/ml of ivermectin used  s_1.4 = (exp(-iv_aa_model$m$getPars()[2]*(a-iv_aa_model$m$getPars()[1])))/(1+exp(-iv_aa_model$m$getPars()[2]*(a-iv_aa_model$m$getPars()[1])))  load("sp_aa_model.Rdata")  a = log2(20) # 20ug/ml of spirotetramat used  s_20 = (exp(-sp_aa_model$m$getPars()[2]*(a-sp_aa_model$m$getPars()[1])))/(1+exp(-sp_aa_model$m$getPars()[2]*(a-sp_aa_model$m$getPars()[1])))  b = log2(24) # 24ug/ml of spirotetramat used  s_24 = (exp(-sp_aa_model$m$getPars()[2]*(b-sp_aa_model$m$getPars()[1])))/(1+exp(-sp_aa_model$m$getPars()[2]*(b-sp_aa_model$m$getPars()[1])))  # fitness of genotypes under selection  spir_wAA = fecundity["SR42_20"]/fecundity["PD4792_20"] * 1 # resistance strain fitness only affected by baseline fecundity, not by compound selection  spir_20_waa = 1 * s_20  spir_24_waa = 1 * s_24  iver_wAA = fecundity["JD608_22.5"]/fecundity["PD4792_22.5"] * 1  iver_waa = 1 * s_1.4 # susceptible strain fitness only affected by compound selection, no baseline fecundity  # set simulation parameters  # number of generations  if(grepl("spirotetramat", setup, fixed=TRUE)){  t = 7  } else {  t = 5  }  # number of simulation repeats  reps = 100  # population size  k = 7500  # initial resistant allele frequency  if(grepl("spirotetramat", setup, fixed=TRUE)){  fA0 = 0.05  } else {  fA0 = 0.5  }  # save data from simulation  fA_reps = matrix(fA0,t+1,reps)  # simulation  for(i in 1:reps){  for(j in 1:t){  # set up at gen 1  if(j==1){  genotypes = matrix(0,t+1,2)  colnames(genotypes) = c("fAA","faa") # only homozygote resistant and homozygote susceptible individuals  genotypes[1,] = c(fA0,1-fA0) # set up genotype frequenies    # set genotype fitness  if(setup=="spirotetramat_moderate"){  wAA = spir_wAA  waa = spir_20_waa  } else if(setup=="spirotetramat_high"){  wAA = spir_wAA  waa = spir_24_waa  } else if(setup=="ivermectin_treatment"){  wAA = iver_wAA  waa = iver_waa  }  }    # genotype frequencies  fAA = genotypes[j,1]  faa = genotypes[j,2]    # survival frequencies #this is where the fitness cost shows up in the model  sAA = wAA*fAA  saa = waa*faa    # zygote frequencies  zAA = sAA  zaa = saa  # proportional zygote frequencies  wm = zAA + zaa  pAA = zAA/wm  paa = zaa/wm    # stochastic sampling  pset = c(pAA,paa)  qset = rmultinom(1,k,prob=pset)  pAA = qset[1]/k  paa = qset[2]/k    # allele frequencies  pA = pAA  pa = paa    # save allele frequencies to main matrix  fA_reps[j+1,i] = pA    # update genotype frequencies  genotypes[1+j,1] = pAA  genotypes[1+j,2] = paa    }  }  # plot allele frequencies across generations, compare with in vivo model data  fA_reps = data.frame(fA_reps)  fA_reps = melt(fA_reps)  fA_reps$generation = seq(0,t)  temp_data = read_excel("0031 spir_in_vivo_data.xlsx")  trt20 = temp_data[temp_data$condition=="moderate",]  trt24 = temp_data[temp_data$condition=="high",]  temp_data = read_excel("0032 iver_in_vivo_data.xlsx")  trt = temp_data[temp_data$condition=="treatment",]  if(setup=="spirotetramat_moderate"){  df = trt20  } else if(setup=="spirotetramat_high"){  df = trt24  } else if(setup=="ivermectin_treatment"){  df = trt  }  p = ggplot(data = fA_reps, aes(x = generation,y=value,group=variable,colour=variable))+  geom_line()+  theme_classic()+  theme(legend.position="none",  axis.text=element_text(size=12),  axis.title=element_text(size=15))+  scale_colour_manual(values = unname(viridis(100)))+  ylim(c(0,1))+  scale_x_continuous(breaks = seq(0,t))+  geom_point(inherit.aes = F, data = df, aes(x=generation,y=frequency),size=2,alpha=0.5)+  labs(x="Generation",y="Resistance allele frequency")  print(p) |
| --- |

**S4a. Selection coefficient fitted from *in vivo* model of spirotetramat selection at 20 µg/ml.**


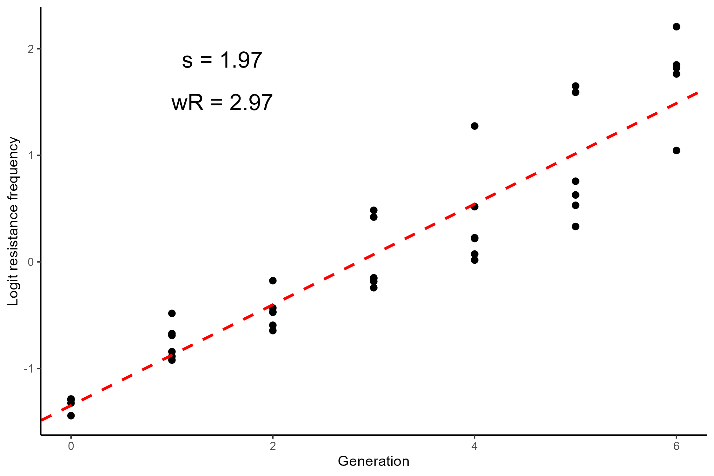


**S4b. Selection coefficient fitted from *in vivo* model of spirotetramat selection at 24 µg/ml.**


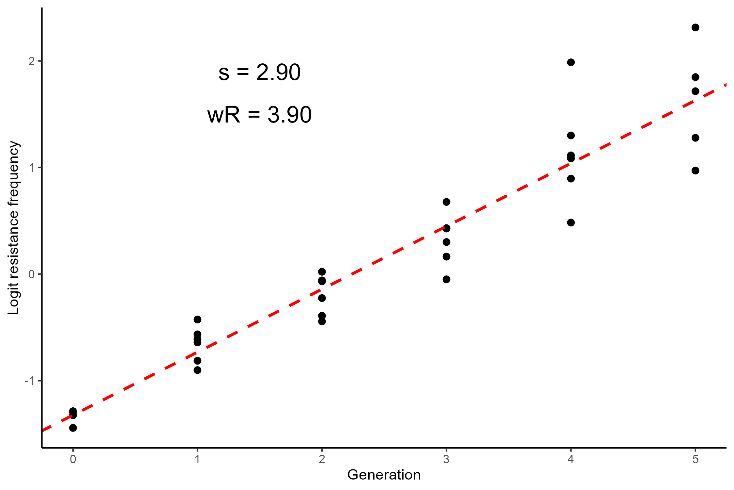


**S4c. Selection coefficient fitted from *in vivo* model of ivermectin selection at 1.4 ng/ml**


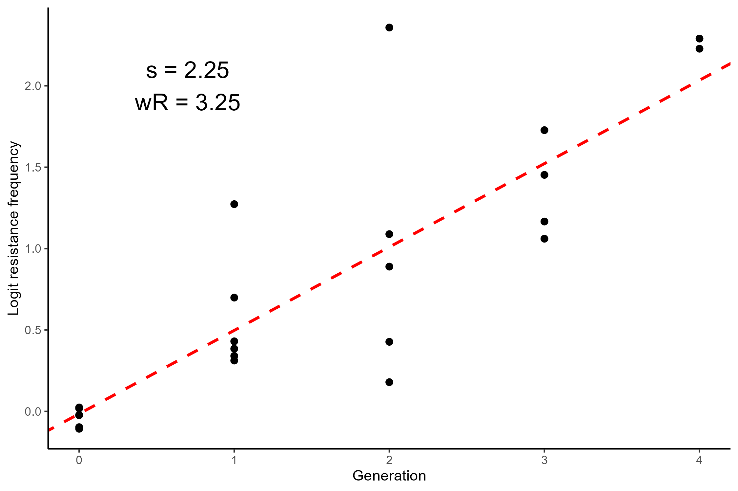

Supplement: Supplementary materials [file EMS213312-supplement-Supplementary_materials.docx]
